# CYP19A1 Silencing Inhibits Cell Proliferation and Endoplasmic Reticulum Stress in Stomach Adenocarcinoma

**DOI:** 10.32604/or.2025.062250

**Published:** 2025-09-26

**Authors:** Yi Jin, Zexing Shan, Fan Yang, Xinwen Fan, Jie Lin, Zeqing Huang, Xudong Zhu

**Affiliations:** 1 Department of Anesthesiology, Cancer Hospital of Dalian University of Technology, Cancer Hospital of China Medical University, Liaoning Cancer Hospital & Institute, Shenyang, 110042, China; 2 Department of Gastric Surgery, Cancer Hospital of Dalian University of Technology, Cancer Hospital of China Medical University, Liaoning Cancer Hospital & Institute, Shenyang, 110042, China; 3 Department of General Surgery, Cancer Hospital of Dalian University of Technology, Cancer Hospital of China Medical University, Liaoning Cancer Hospital & Institute, Shenyang, 110042, China; 4 Liaoning Provincial Key Laboratory of Precision Medicine for Malignant Tumors, Liaoning Cancer Hospital & Institute, Shenyang, 110042, China

**Keywords:** ER stress, stomach adenocarcinoma, prognostic prediction signature, nomogram, Cytochrome P450 Family 19 Subfamily A Member 1 (CYP19A1)

## Abstract

**Background:**

As a major histopathological subtype of gastric cancer (GC), stomach adenocarcinoma (STAD) is an important malignant tumor in the digestive system. Increasing evidence also indicates that endoplasmic reticulum (ER) stress plays a pivotal role in the pathogenesis and progression of GC. Therefore, this study aims to screen and identify vital ER stress-related genes that could contribute to the malignant development and poor prognosis for STAD.

**Methods:**

A novel ER stress-related risk score signature was developed employing machine learning techniques. Then, a prognostic prediction nomogram was also built based on the clinicopathological characteristics and the risk score signature. The tumor immune microenvironment characteristics and pathway enrichment analysis in different risk groups were also explored. Furthermore, through the single-cell RNA sequencing (scRNA-seq) analysis, the study highlighted Cytochrome P450 Family 19 Subfamily A Member 1 (CYP19A1) as the pivotal research target and detected its effect on cell proliferation by 3-(4,5-dimethylthiazol-2-yl)-2,5-diphenyl tetrazolium bromide (MTT) and the expression of ER stress-related genes by RT-qPCR in STAD.

**Results:**

Based on the evaluation of five screened key ER stress-related genes (*AKR1B1*, *SERPINE1*, *ADCYAP1*, *MATN3*, *CYP19A1*), our ER stress-related risk score signature offers a novel approach for assessing STAD prognosis hazards. The novel prognostic prediction nomogram based on the signature also accurately predicted the survival outcomes of patients with STAD. Furthermore, the expression of *CYP19A1* is significantly higher in STAD tissues than in normal tissues. High expression of *CYP19A1* was related to a poor survival outcome for patients with STAD. Besides, compared to normal gastric epithelial cells, the expression of *CYP19A1* was significantly higher in STAD cell lines. Silencing the expression of *CYP19A1* significantly inhibited the cell proliferation ability and decreased the expression of ER stress-related genes, including *ATF4*, *DDIT3* and *XBP1* in STAD.

**Conclusions:**

In conclusion, our study developed a novel prognosis prediction signature and identified the novel diagnostic and therapeutic target CYP19A1 for patients with STAD.

## 1 Introduction

Gastric cancer (GC) is the fifth most common malignant tumor and the third leading cause of cancer-related deaths globally, and will cause nearly 800,000 deaths in 2020 [1]. It is estimated that the incidence of GC will be 26,890, and deaths number will be 10,880 in the United States in 2024 [2]. Furthermore, the high mortality caused by the poor prognosis of GC exhausted the medical resources both in China and the United States [2,3]. Despite recent studies showing a downward tendency in the incidence and mortality of GC, the 5-year survival rate for GC remains pessimistic among all kinds of cancers [4–6]. GC mainly manifests as stomach adenocarcinoma (STAD), which includes various subtypes such as tubular adenocarcinoma, papillary adenocarcinoma, and poorly cohesive carcinoma. This diversity in clinical presentation also makes the prognosis of STAD patients highly variable [7–10]. Studies have shown that neoadjuvant chemotherapy results in fewer complications and long-term survival opportunities for patients with STAD [8]. For specific types of advanced GC, such as the intestinal subtype, patients can achieve pathological regression after receiving neoadjuvant chemotherapy [11]. Therefore, it is critical to identify a new prognostic trait that can accurately predict the prognosis of patients with STAD and guide treatment decisions.

As the largest organelle in eukaryotic cells, the endoplasmic reticulum (ER) plays a crucial role in protein synthesis, processing and transport [12]. However, the unfolded protein response (UPR) was triggered by cellular stress conditions such as glucose deficiency, hypoxia, and imbalanced calcium levels [13]. In order to alleviate cellular damage caused by UPR, Inositol Requiring Enzyme 1 (IRE1), activating transcription factor 6 (ATF6) and protein kinase R-like ER kinase (PERK) pathways induce complex biological processes to promote cell adaptation [14]. Conversely, sustained ER stress, especially in secretory cells with persistently activated UPR, can lead to cell death [15,16]. However, in tumor cells, sustained ER stress and dysregulated expression of ER stress-related genes (ERSRGs) accelerate the malignant development of tumors [17]. For instance, in colon cancer, Cancerous inhibitor of protein phosphatase 2A (CIP2A) can be upregulated by ATF6 and contributes to poor prognosis [18]. IRE1a-X-Box Binding Protein 1 (XBP1) pathway has also been proven by activating c-MYC signaling pathway to support the growth of prostate cancer cells [19]. Knockdown of XBP1 enhances immunotherapeutic efficacy of antibodies blocking programmed death protein 1 (PD1) and cytotoxic T lymphocyte-associated antigen 4 (CTLA4) in melanoma [20]. These results indicated that the ER stress was extensively involved in the process of tumor malignant development. As for STAD, ERSRGs also play a crucial role in promoting tumor growth and potentially leading to the discovery of novel therapeutic targets [21]. For instance, as a major ER chaperone, Glucose-Regulated Protein 78 (GRP78) is overexpressed in GC, which could promote proliferation and inhibit apoptosis of STAD cells [22]. Pro-apoptotic protein C/EBP homologous protein (CHOP), as a key factor in the PERK pathway during ER stress, is significantly down-regulated at both mRNA and protein levels in STAD tissues and negatively correlated with clinical stages and survival outcomes [23]. Therefore, a comprehensive analysis of ERSRGs in STAD may provide valuable evidence for the clinical diagnosis and therapy [24]. The Cytochrome P450 Family 19 Subfamily A Member 1 (*CYP19A1*) gene encodes an enzyme that belongs to the cytochrome P450 superfamily [25]. This enzyme is responsible for aromatase expression and estrogen synthesis, regulated by hormones, cytokines, and immune modulators under both normal and pathophysiological conditions. Many studies have shown that CYP19A1 can regulate endoplasmic reticulum stress by encoding estrogen, which also includes the initiation and progression of solid cancers [26–28].

In this study, owing to screening and identifying vital ER stress-related genes that could contribute to malignant development and poor prognosis for STAD, we first built a novel ER stress-related risk score signature. Then, a prognostic nomogram was built and validated for patients with STAD. Furthermore, we explored the tumor immune microenvironment characteristics in different risk groups and detected the distribution of five screened ERSRGs in the GC tumor microenvironment by Single-cell RNA sequencing (scRNA-seq) analysis. At last, we selected *CYP19A1* as the research target and found that CYP19A1 knockdown may inhibit STAD cells’ proliferation and decrease the expression of ERSRGs, including Activating Transcription Factor 4 (*ATF4*), DNA Damage Inducible Transcript 3 (*DDIT3*) and *XBP1*. In conclusion, our study developed a novel prognosis prediction signature and provided novel diagnostic and therapeutic targets for patients with STAD.

## 2 Materials and Methods

### 2.1 Data Acquisition

Transcriptome data were obtained from the Cancer Genome Atlas (TCGA) (https://www.cancer.gov/ccg/research/genomesequencing/tcga (accessed on 10 July 2025)) database for 407 STAD patients, including 32 normal stomach and 375 STAD samples (The clinical information of 355 patients was available and used in this study). ERSRGs were acquired from the Genecard website (https://www.genecards.org/). Only genes with a relevance score greater than 7 were eligible for inclusion, and 1242 relevance score were enrolled. TCGA-STAD clinical information was also obtained from the TCGA database. The “limma” package (version 3.58.1) was employed to recognize differentially expressed genes (DEGs). For the GSE84437 dataset, it was downloaded from the Gene Expression Omnibus (GEO) database (http://www.ncbi.nlm.nih.gov/geo/(accessed on 10 July 2025)). Patients’ characteristics are shown in Table 1. In addition, the GC scRNA-seq dataset (GSE167297) was also sourced from the GEO database, which involved the analysis of surgically dissected superficial and deep layers of five diffuse-type GC samples along with matched normal tissue samples. Matrix data and clinical data information were extracted using R software (version 4.3.1). Patients’ characteristics are shown in Table 2.

**Table 1:** Clinicopathological information of TCGA-STAD and GSE88437

TCGA		GSE88437	
Variables	No. of samples	Variables	No. of samples
**Sex**		**Sex**	
Male	228	Male	296
Female	127	Female	137
**Age at diagnosis**		**Age at diagnosis**	
≤65	159	≤65	283
>65	193	>65	150
Unknown	3	Unknown	0
**Stage**		**Stage**	
I	48	I	/
II	111	II	/
III	146	III	/
IV	36	IV	/
Unknown	14	Unknown	/
**Survival status**		**Survival status**	
Alive	211	Alive	224
Dead	144	Dead	209

**Table 2:** Clinicopathological information of five patients with diffuse-type gastric cancer

Sample	Age	Sex	Resection	Lymph nodes	Sample type	Tissue
Patient 1	65	Female	Total	12/46	23,060 single cells	Gastric cancers
Patient 2	69	Male	Subtotal	1/36	23,060 single cells	Gastric cancers
Patient 3	60	Male	Subtotal	2/55	23,060 single cells	Gastric cancers
Patient 4	46	Female	Subtotal	0/62	23,060 single cells	Gastric cancers
Patient 5	59	Male	Subtotal	9/47	23,060 single cells	Gastric cancers

#### 2.1.1 The Construction and Validation of the ERSRGs Risk Score Signature

DEGs were identified using the “limma” R package (version 3.58.1) based on the data of TCGA-STAD. Then, a total of 1242 ERSRGs underwent differential analysis, adhering to a threshold criterion of *p* value < 0.05 and a false discovery rate below 0.05 (FDR ≤ 0.05). By the intersection of TCGA-DEGs, ERSRGs and GSE84437 gene sets, we obtained 873 selected ERSRGs. To identify differentially expressed ERSRGs that were associated with overall survival (OS), univariate Cox regression analyses were performed using the “Survival” R software package (version 3.7-0). Then, these genes whose *p* values < 0.05 were included in the least absolute shrinkage and selection operator (LASSO) regression analyses by using the “glmnet” R package (version 4.1-8). The penalty parameter (λ) and the minimum criterion for the gene expression level were also determined. The risk scoring equation was: risk score = (exprgene1 × Coefgene1) + (exprgene2 × Coefgene2) + ⋯ + (exprgene5 × Coefgene5). According to the risk score value, survival time and survival state, the “survminer” package (version 0.4.3) was used to calculate the optimal cut-off value of the risk score. The patients within the TCGA-STAD cohort were divided into a high-risk group and a low-risk group. Then, “survival” R packages were used to depict Kaplan-Meier (K-M) curves. Furthermore, we also evaluated the sensitivity of our novel risk score signature using receiver operating characteristic (ROC) curves. Besides, the GSE84437 dataset was used as the validation cohort.

#### 2.1.2 Functional Enrichment Analysis

The cutoff values of the ERSRGs risk score signature separated TCGA-STAD training cohort into high-risk and low-risk groups, then DEGs were conducted between two groups using “limma” package, The screening criteria were set as |log fold change (FC)| > 1.0 and *p* < 0.05. The Gene Ontology (GO) and Kyoto Encyclopedia of Genes and Genomes (KEGG) based on different groups of DE-ERSRGs were then fully explored using the “clusterProfiler” R package (version 4.10.1).

#### 2.1.3 Immune Cell Infiltration and Immune Checkpoint Genes Expression Analysis

The immune cell composition was identified by single sample gene set enrichment analysis (ssGSEA) method packaged with GSVA (version 1.50.5), and the enrichment level of immune cells in the tumor microenvironment was evaluated by the gene expression level in a single tumor sample. Additionally, to determine the relationships between these ERSRGs computed risk scores and immune cell abundance, immune function, and immune checkpoints, we compared the immune landscape of STAD between the two risk groups using the “limma” R package. Simultaneously, the R package “limma” was also used to analyze the differences of immune checkpoint genes in the TCGA-STAD and GSE84437 databases between different risk groups.

#### 2.1.4 Formulation and Evaluation of the Nomogram

Utilizing the R packages “survival” and “regplot” (version 1.1), a nomogram was meticulously constructed to forecast patients’ survival at 1-, 3-, and 5-year intervals. Calibration curves and nomogram ROC curves were constructed using the “rms” R package (version 6.8-1) to assess the accuracy of the nomogram in predicting 1-, 3-, and 5-year survival in patients with STAD.

#### 2.1.5 scRNA-seq Data Analysis

“Seurat” package (version 5.1.0) was employed for the processing of scRNA-seq data. Fewer than 50 genes expressed in cells, as well as the expression of genes which is less than three cells, were systematically excluded from the data set. After the initial data preprocessing phase, dimension reduction was performed, followed by a comprehensive t-Distributed Stochastic Neighbor Embedding (t-SNE) cluster analysis.

#### 2.1.6 Cell Culture and Cell Transfection

RPMI-1640 medium supplemented with 10% FBS (Procell Life Science & Technology Co., Ltd., Wuhan, China) was used in culturing GES-1, HGC27, MKN45 and AGS cell lines (Cell Bank of the Chinese Academy of Sciences, Shanghai, China). Specifically, the GES-1 cell line is derived from normal human gastric epithelial cells, while HGC27, MKN45, and AGS are derived from human STAD cells. All these cells have been confirmed to there is not mycoplasma contamination and have undergone authentication (STR) identification. The medium was maintained at 37°C in a 5% CO_2_ atmosphere.

For transfection purposes, cells were treated with 100 nM siRNA (Mbi Tech, Shenyang, China). The cell number is 500,000/well, and the used transfection reagent is Lipo8000™ (Beyotime Biotechnology, C0533, Shanghai, China).

Small interfering RNA (siRNA) sequences targeting CYP19A1 were listed as follows: si1-CYP19A1: CUUUGGGAAUAAUAAUCGUUCAGGA, si2-CYP19A1: UCCUGAACGAUUAUUAUUCCCAAAG, si-NC: UUCUCCGAACGUGUCACGUTT.

#### 2.1.7 Real-Time Quantitative Polymerase Chain Reaction (RT-qPCR) Analysis

Using Trizol Up reagent (TransGen Biotech, Beijing, China), the total RNA was extracted from gastric epithelial cells and GC cell lines. The RNA concentration and purity were assessed using a Nanodrop (Thermo Fisher Scientific, Waltham, MA, USA), focusing on the 260/280 absorbance ratio. For RT-qPCR, samples were treated with 2× TB Green qPCR mix (Takara, Beijing, China), and primer sequences were obtained from SANGON Biotech (Shanghai, China). In this assay, an endogenous control, GAPDH mRNA, was used to normalize relative mRNA expression levels, which were calculated using the 2^−ΔΔCT^ method. The specific primer sequences employed were presented in Table 3. The experiments were independently repeated three times.

**Table 3:** The sequences of the primers

Gene names	Species	Sequence	
CYP19A1	*Homo sapiens* (human)	Forward	5^′^-ATGAAAGCTCTGTCAGGCCC-3^′^
		Reverse	5^′^-TCAACACGTCCACATAGCCC-3^′^
ATF4	*Homo sapiens* (human)	Forward	5^′^-TCTGCCCGTCCCAAACCTTAC-3^′^
		Reverse	5^′^-CCTGCTCCGCCCTCTTCTTC-3^′^
DDIT3	*Homo sapiens* (human)	Forward	5^′^-ACCCTGCTTCTCTGGCTTGG-3^′^
		Reverse	5^′^-CCTTGGTCTTCCTCCTCTTCCTC-3^′^
XBP1	*Homo sapiens* (human)	Forward	5^′^-TCCGCAGCACTCAGACTACG-3^′^
		Reverse	5^′^-GGGTCCAAGTTGTCCAGAATGC-3^′^
GAPDH	*Homo sapiens* (human)	Forward	5^′^-TCAAGATCATCAGCAATGCC-3^′^
		Reverse	5^′^-CGATACCAAAGTTGTCATGGA-3^′^

#### 2.1.8 3-(4,5-Dimethylthiazol-2-yl)-2,5-Diphenyl Tetrazolium Bromide (MTT) Assay

MTT solution (M8180, Solarbio Science & Technology, Beijing, China) of 20 μL was added to each well of a 96-well plate with 2000 cells per well in order to assess cell proliferation. Three replicates were established for each condition. After incubation at 37°C and 5% CO_2_ for 24 h to induce cell adhesion, the medium and MTT reagent were carefully removed from the wells. Subsequently, 150 μL dimethyl sulfoxide (DMSO) was added to each well and stirred at 100 rpm (Allsheng MB100-4A Instruments Co., Ltd., Hangzhou, China) at 37°C for 10 min to promote dissolution. Cell viability was detected by measuring absorbance at 570 nm using a microplate reader (Tecan, i-control-infinite F50, Männedorf, Switzerland). Repeat each experimental condition three times to ensure the reliability and accuracy of the results.

### 2.2 Data Analysis

Statistical analyses of data were conducted using the R software, version 4.3.1. Differences of categorical and continuous variables were compared by Chi-square and Student’s *t*-tests. For cellular clustering of single-cell sequencing, both the t-SNE method and principal component analysis (PCA) were applied as dimensionality reduction techniques. The comparative analysis of immune checkpoint gene expression levels between the high-risk and low-risk groups was conducted utilizing the Wilcoxon test. Statistical significance was established at levels of *p < 0.05, **p < 0.01, ***p < 0.001 and ****p < 0.0001, as determined by two-tailed statistical tests.

## 3 Results

### 3.1 Identification of Prognostic ERSRGs

**Figure 1:**
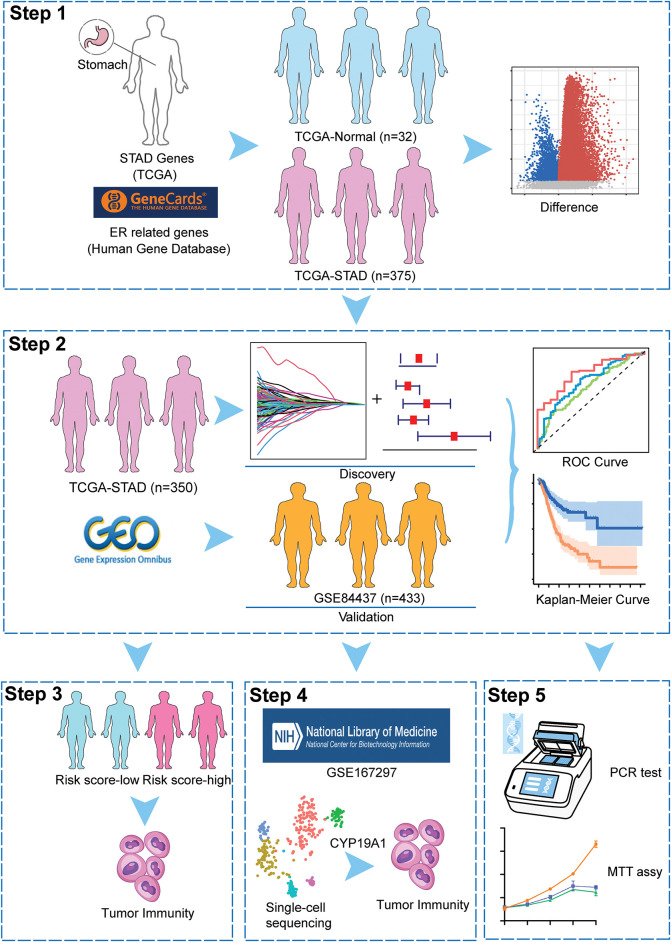
The overall schematic design of this work

**Figure 2:**
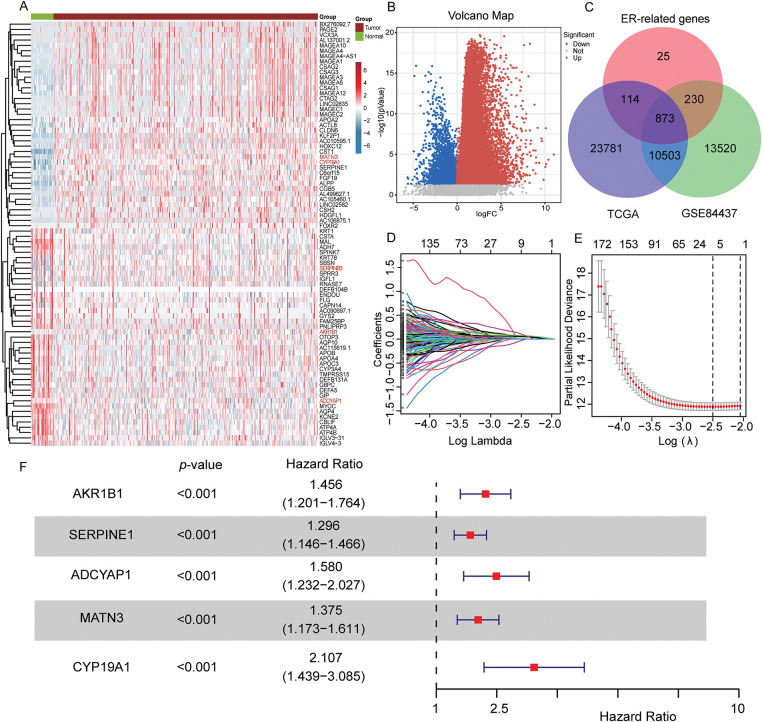
Construction of the prognostic risk score signature. (**A,B**) Heatmap and volcano map of TCGA-STAD shows different expressed genes between normal stomach and STAD samples; (**C**) Intersecting genes associated with TCGA-STAD-DEGs, GSE84437 (mRNA-array) databases and ERSRGs; (**D**) LASSO coefficient profiles of the 873 genes in the TCGA-STAD dataset; (**E**) LASSO (lambda) choice of the optimal parameters in the signature; (**F**) Hazard ratio of the five selected genes

The overall schematic design of this work is exhibited in Fig. 1. Firstly, compared with normal samples, DEGs were extracted from the TCGA-STAD cohort (*p* < 0.05, Fig. 2A,B). Then, the intersection of TCGA-STAD-DEGs, GSE84437 and 1242 ERSRGs was performed and 873 ERSRGs were obtained by Venn diagram (Fig. 2C, Supplementary Tables S1–S3). These 873 overlapping genes were subjected to univariate Cox regression and K-M analysis in the TCGA-STAD dataset, resulting in 65 genes considered to be OS-related genes (Supplementary Table S4). In order to avoid the risk characteristics of the fitting problem, we used the TCGA-STAD data to perform LASSO regression analysis (Fig. 2D,E). According to the respective amount of gene expression and the regression coefficient, a signature including five prognostic ERSRGs were built, as shown in the following: Risk Score = 0.0599793913280681 ∗ Aldo-Keto Reductase Family 1 Member B1 (*AKR1B1*) + 0.071044832660098 ∗ Serpin Family E Member 1 (*SERPINE1*) + 0.00850483124114563 ∗ Adenylate Cyclase Activating Polypeptide 1 (*ADCYAP1*) + 0.0492099441258326 ∗ Matrilin 3 (*MATN3*) + 0.0692156221207668 ∗ *CYP19A1* (Fig. 2E, Supplementary Table S5). Fig. 2F shows the Hazard Ratio of the five prognostic ERSRGs.

### 3.2 Evaluating the ERSRGs Risk Score Signature

**Figure 3:**
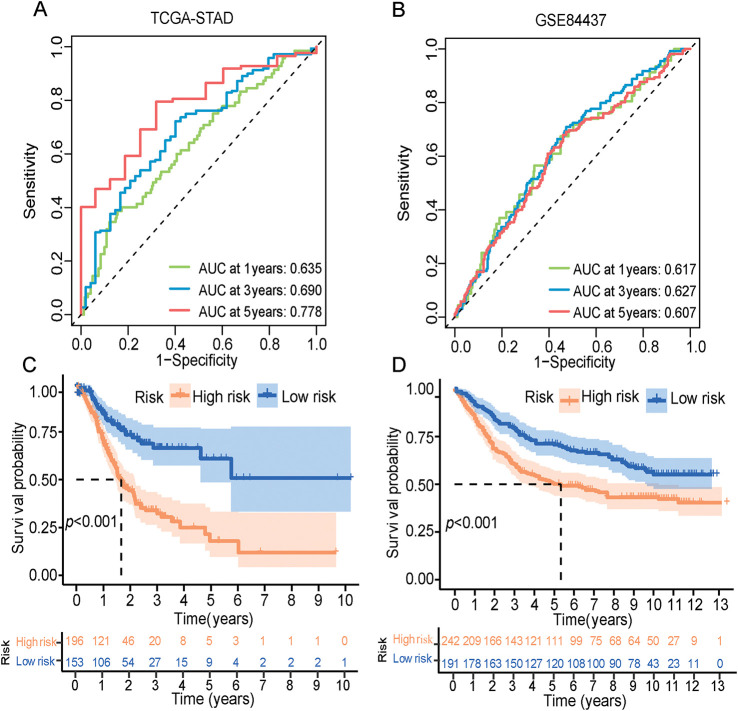

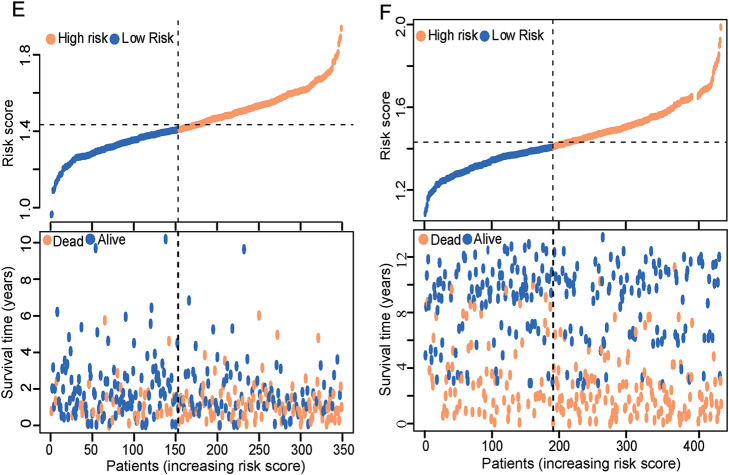
ERSRGs risk score signature prediction capability assessment and pathway enrichment analysis. (**A**) Evaluation of the prognostic prediction capability of the ERSRGs risk score signature using the ROC curve based on the TCGA-STAD data; (**B**) Evaluation of the prognostic prediction capability of the ERSRGs risk score signature using the ROC curve based on the GSE84437 data; (**C**) K-M survival curve of the different risk groups based on the TCGA-STAD data; (**D**) K-M survival curve of the different risk groups based on the GSE84437 data; (**E**) Risk score distribution of the patients based on the TCGA-STAD data; (**F**) Risk score distribution of the patients based on the GSE84437 data

The ERSRGs risk scores of patients in the training queue (TCGA-STAD data sets) and validating queue (GSE84437 dataset) were calculated according to the above formula, and these patients were divided into high-risk and low-risk groups according to the calculated risk scores. The predictive efficiency of ER stress-related risk features was evaluated using a time-varying ROC curve. The Area Under the Curve (AUC) for 1-, 3- and 5-year OS in the TCGA data set were 0.635, 0.690 and 0.778, respectively, and the GSE84437 data set were 0.617, 0.627 and 0.607, respectively (Fig. 3A,B). An intuitive advantage of longer OS and lower mortality in the low-risk group over the high-risk group was demonstrated by the K-M survival curves of the training set and the GSE84437 validating set (training set: *p* < 0.001, validation set: *p* < 0.001, Fig. 3C,D). The high- and low-risk groups were able to be successfully separated by risk scores, and the number of deaths in the high-risk group increased with the risk scores (Fig. 3E,F, Supplementary Tables S6 and S7). The risk scores can also significantly differentiate between high- and low-risk groups of patients across different ages, sexes, and clinical stages (Fig. S1A–C). Furthermore, compared to similar GC models, the area under the ROC curve for 1-year survival is 0.635, which is not superior to that of Models 1 and 2 (Fig. S1D). However, the areas under the ROC curves for 3-year and 5-year survival are 0.69 and 0.778, respectively, which are quite higher than those of Models 1 and 2 (Fig. S1E,F) [29,30].

### 3.3 Function Enrichment Analysis Based on ERSRGs Risk Score Signature

Meanwhile, we further explored the expression level of ER stress markers in different risk groups and found that most of them were more highly expressed in the high-risk group, indicating that the ER stress in the high-risk group was significantly stronger than that in the low-risk group (Fig. S2A,B). In consideration of the potential differences in biological processes and functional pathways among different risk groups in the TCGA-STAD cohort, we also performed pathway enrichment analysis for individual investigations. The primary GO terms enriched included “extracellular matrix organization”, “extracellular structure organization” in biological process (BP), “collagen-containing extracellular matrix”, “endoplasmic reticulum lumen” in cellular component (CC), and “extracellular matrix structural constituent”, “glycosaminoglycan binding” in molecular function (MF), which indicating that STAD cells in the high-risk group may be more active in extracellular matrix (ECM) regulation and cell-cell interactions. Furthermore, in the KEGG pathway, the ECM receptor interaction pathway, the PI3K/AKT signaling pathway and other cancer-related pathways were also identified (Fig. S2C,D). The GSEA results also illustrated that the conspicuously KEGG pathway in high-risk group patients was “ECM receptor interaction”, and “cell adhesion molecules cams” (Fig. S2E,F, Supplementary Tables S8–S10).

### 3.4 Construction and Validation of a Novel Prognostic Nomogram for Patients with STAD Partly Based on ERSRGs Risk Score Signature

**Figure 4:**
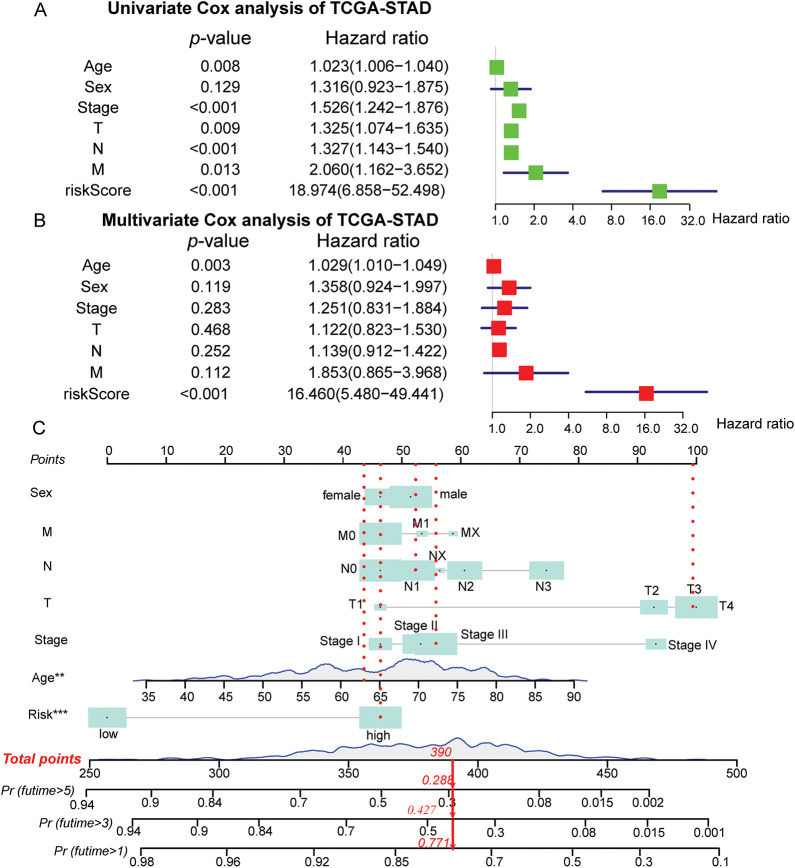

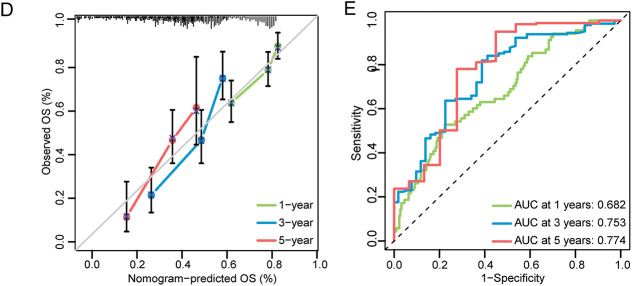
Construction and validation of the prognostic nomogram in the TCGA-STAD cohort. (**A**) Forest maps of univariate Cox regression analysis; (**B**) Forest maps of multivariate Cox regression analysis; (**C**) Prognostic nomogram was plotted according to class, age, sex, T, N, M status, and risk score; (**D**) The calibration chart of the nomogram; (**E**) ROC curves for 1-, 3-, and 5-Year of the nomogram. **p < 0.01, ***p < 0.001

The study further constructs and validates a novel prognostic nomogram based on the risk score signature and clinicopathological characteristics of patients with STAD. Univariate Cox analysis was employed to demonstrate that age, sex, grade, and risk score signature were significantly related to OS (Fig. 4A). Subsequent multivariate Cox regression analysis further presented that besides age and stage, risk score signature was also significantly associated with OS, which indicated that the risk score signature may serve as an independent prognostic predictor of OS for patients with STAD (Fig. 4B). Furthermore, by integrating the ERSRGs risk score, age, sex, and staging status, we constructed a novel prognostic nomogram based on the TCGA dataset, which could predict 1-, 3-, and 5-year OS conditions of patients with STAD. In this nomogram, each feature was assigned a score based on its OS risk contribution. Notably, both age and risk stratification retained independent prognostic value following multivariable adjustment (Fig. 4C, Supplementary Table S11). The calibration curves also demonstrated significant concordance between forecast survival times and actual survival times for 1-, 3-, and 5-year OS rates in the TCGA cohort (Fig. 4D). In addition, we evaluated the predictive performance of this prognostic model for 1-, 3-, and 5-year OS using ROC curves, yielding AUCs of 0.682, 0.753, and 0.774, respectively (Fig. 4E). Such findings illustrated that this nomogram could accurately predict the survival outcomes for patients with STAD and can be used for precise clinical prognosis prediction.

### 3.5 The Deep Analysis of Tumor Immune Microenvironment Characteristics in Different Risk Groups

**Figure 5:**
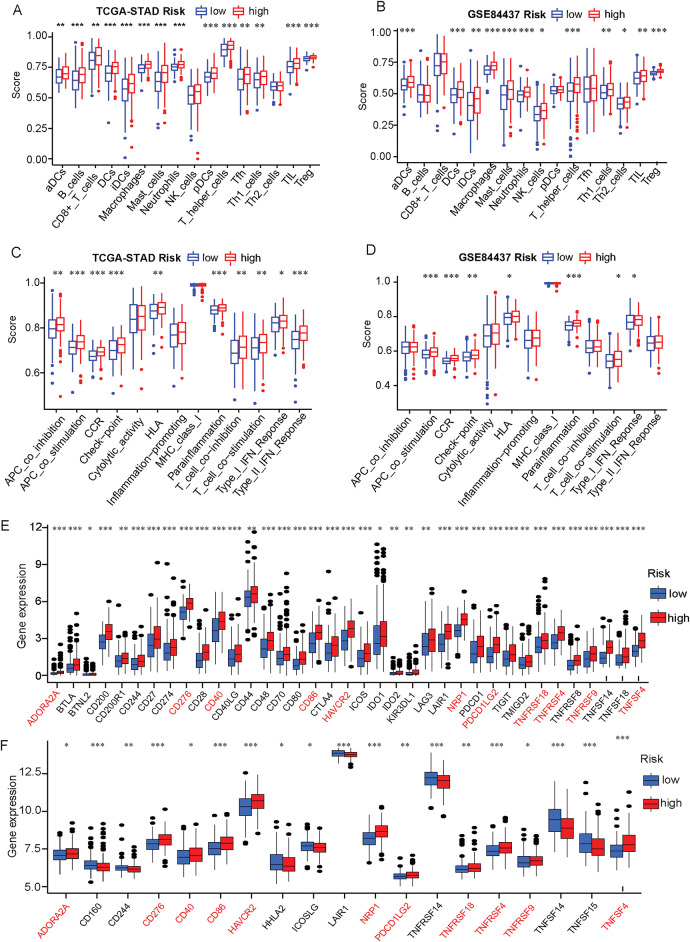
Immune status and immune checkpoint analysis in the TCGA-STAD and GSE84437 datasets in the different risk groups. (**A**) The expression level of immune cells was compared between high- and low-risk groups based on the TCGA-STAD data; (**B**) The expression level of immune cells was compared between high- and low-risk groups in the GSE84437 dataset; (**C**) Immune function scores were compared between high- and low-risk groups in the TCGA-STAD cohort based on the ssGSEA algorithm; (**D**) Immune function scores were compared between high- and low-risk groups in the GSE84437 dataset based on the ssGSEA algorithm; (**E**) The expression level of immunization checkpoint genes in the different risk groups based on the TCGA-STAD data; (**F**) The expression level of immunization checkpoint genes in the different risk groups based on the GSE84437 data. *p < 0.05, **p < 0.01, ***p < 0.001

Firstly, significant differences in the level of infiltrating immune cells and tumor immune microenvironment characteristics were explored in the different risk groups (Fig. S3, Supplementary Table S12). Based on the ssGSEA methodology, significantly higher immune cells infiltration levels, including activated Dendritic Cells (aDCs), Dendritic Cells (DCs), Immature Dendritic Cells (iDCs), macrophages, mast cells, neutrophils, Type 1 T helper (Th1), Tumor-Infiltrating Lymphocytes (TIL), and Tregs, were found in the high-risk patient group based on both the TCGA cohort and GSE84427 cohort. Other immune cells such as B cells, CD8^+^ T cells, NK cells, plasmacytoid dendritic cells (pDCs), T follicular helper cells (Tfh) and Type 2 T helper (Th2) cells also had significantly higher levels of infiltration in high-risk patients group based on the TCGA cohort (Fig. 5A,B Supplementary Table S13). In terms of immune function, “Antigen-Presenting Cell (APC) co-stimulation”, “C Chemokine Receptor (CCR)”, “human leukocyte antigen (HLA)”, “parainflammation”, “T-cell co-stimulation”, and “type I IFN response” were also significantly associated with high-risk scores, both in TCGA and GSE84437 cohorts (Fig. 5C,D Supplementary Table S14). With these findings, it can be confirmed that the ERSRGs risk score signature was significantly coherent with the high- and low-risk groups and had good applicability in the TCGA-STAD and GSE84437 datasets.

Meanwhile, owing to many studies have found that the common immune checkpoint molecules tend to be significantly upregulated in malignant tumors and are associated with poor outcomes [31,32]. Therefore, we used the Wilcoxon test to compare the expression level of various immune checkpoint genes in the high-risk and low-risk groups. We found that the expression of 37 immune checkpoint genes in the TCGA-STAD cohort was statistically distinguishable from the ERSRGs high and low risk groups. More to the point, the expression of 37 immune checkpoint genes was positively related to the risk score in the TCGA-STAD cohort (Fig. 5E). Furthermore, 19 immune checkpoint genes were statistically different in the high and low risk groups in the GSE84437 cohort (Fig. 5F, Supplementary Table S15). Furthermore, there were 11 identical immune checkpoint related genes whose expressions were positively correlated with the risk scores in TCGA and GEO databases (Fig. S4).

### 3.6 Distribution of These Five Screened ERSRGs in the GC Tumor Microenvironment Explored by scRNA-seq Analysis

**Figure 6:**
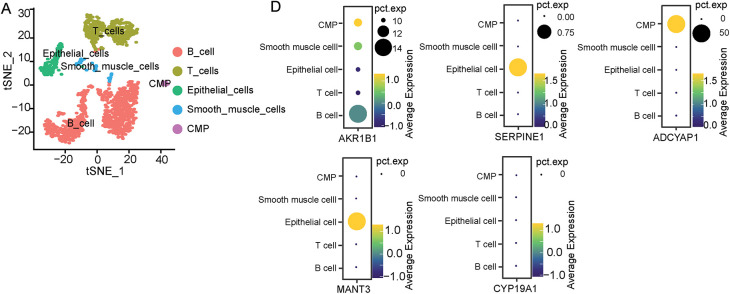

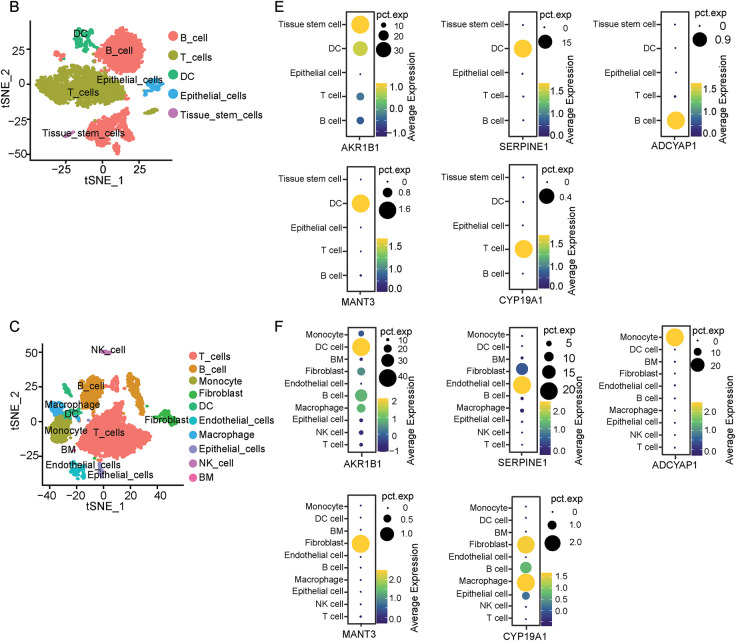
Distribution of five screened ERSRGs in the GC tumor microenvironment explored by scRNA-seq analysis based on GSE167297. (**A**) scRNA-seq cell annotation analysis of normal gastric tissues; (**B**) scRNA-seq cell annotation analysis of superficial GC tissues; (**C**) scRNA-seq cell annotation analysis of deep GC tissues; (**D**) Bubble plots of the distribution of five key genes in single-cell analysis of normal gastric tissues; (**E**) Bubble plots of the distribution of five key genes in single-cell analysis of superficial GC tissues; (**F**) Bubble plots of the distribution of five key genes in single-cell analysis of deep GC tissues

To identify the specific distributions of these five screened ERSRGs and their associations with immune cells, the study analyzed a composite atlas of 23,060 single cells from five diffuse GCs derived from superficial and deep surgical dissections, and matched normal tissues by using the GSE167297 dataset. Cells with fewer than 50 expressed genes and genes present in less than 3 cells were selectively excluded (Fig. S5A–C). Then, an analysis in normal tissue, superficial and deep layers of GC, was conducted to explore the correlations between sequencing depth and gene count. We found significantly positive correlations between sequencing depth and gene count with coefficients of 0.8, 0.87 and 0.88, respectively (Fig. S5D,F). Furthermore, following PCA for dimensionality reduction, cell trajectory analysis and t-SNE clustering analysis were also performed. The study found that in normal gastric tissue, epithelial cells, smooth muscle cells, B cells, T cells and common myeloid progenitor (CMP) were the main cellular composition (Figs. 6A and S6A,B). In superficial layers of gastric cancer tissues, B cells, T cells, DC cells, Epithelial cells, and tissue stem cells were the main cellular compositions (Figs. 6B and S6C,D). In deep layers of gastric cancer tissues, B cells, T cells, DC cells, Monocytes, Fibroblasts, Epithelial cells, Macrophages, Endothelial cells, NK cells and Bone marrow (BM) cells were the main cellular compositions (Figs. 6C and S6E,F). Annotation information was also shown in Supplementary Tables S16–S18.

Furthermore, the study detected the distribution of these five selected genes in normal tissues and tumor tissues. As a result, we found that except *CYP19A1*, the expression of *AKR1B1*, *SERPINE1*, *ADCYAP1* and *MATN3* can be observed in normal gastric tissues (Fig. 6D). However, bubble charts which illustrated gene clustering in the model highlighted that only *CYP19A1* exhibited high expression in various tumor clusters but not in normal gastric tissues (Fig. 6E,F). Considering the high Univariate Cox Hazard Ratio (2.107) and high coefficient value (0.0692), the study ultimately selected *CYP19A1* as the primary gene of interest for the following study.

### 3.7 CYP19A1 Was Highly Expressed in STAD and Its Knockdown Inhibited Tumor Cell Proliferation and ER Stress

**Figure 7:**
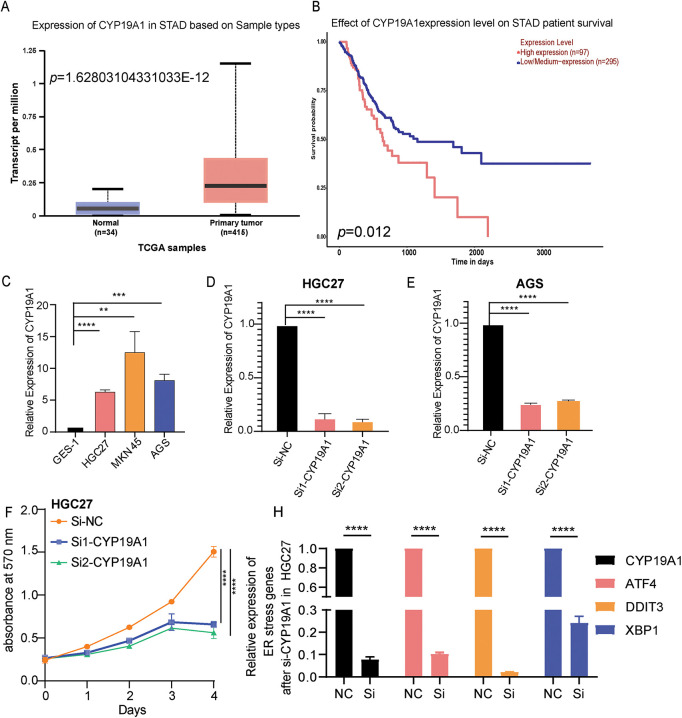

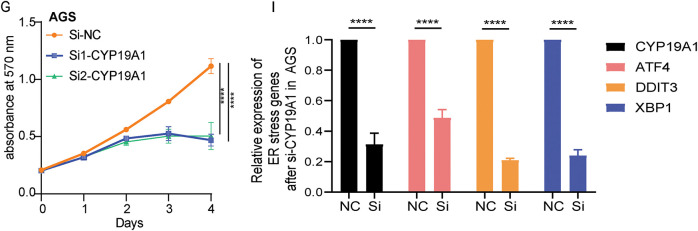
CYP19A1 was highly expressed in STAD and its knockdown inhibited tumor cell proliferation. (**A**) Expression of the CYP19A1 in tumor tissue and their corresponding normal tissues analyzed by the TCGA database; (**B**) The role of CYP19A1 expression in the overall survival of patients with STAD; (**C**) The relative expression of CYP19A1 in GES-1, HGC27, MKN45 and AGS cell lines was measured by RT-qPCR; (**D**,**E**) CYP19A1 mRNA levels were measured in HGC27 and AGS cells after siRNA transfection, detected by RT-qPCR; (**F**,**G**) The effect of CYP19A1 knockdown in the proliferation of HGC27 and AGS cells detected by MTT assay; (**H**,**I**) The expression level of ATF4, DDIT3 and XBP1 was detected in HGC27 and AGS cells after silencing CYP19A1. **p < 0.01, ***p < 0.001, ****p < 0.0001

Firstly, the study detected the expression of *CYP19A1* in STAD tissue and normal tissue in the TCGA-STAD database. The analysis showed that *CYP19A1* had higher expression in tumor tissue than in normal tissue (Fig. 7A). Moreover, high-level expression of *CYP19A1* is also associated with poor survival outcomes (Fig. 7B). GES-1 stomach epithelial cell and HGC27, MKN45, AGS GC cell lines were employed to substantiate the expression of *CYP19A1* via RT-qPCR assay. The results confirmed that the GC cell lines showed significantly higher expression of *CYP19A1* mRNA compared with the gastric epithelial cell lines (**p < 0.01, ***p < 0.001, ****p < 0.0001, Fig. 7C). These findings corroborate our previous database analysis results. In an endeavor to elucidate the impact of CYP19A1 on GC cells, siRNA was employed to selectively downregulate *CYP19A1* expression in HGC27 and AGS cell lines. As depicted in Fig. 7D,E, the transfection with si-CYP19A1 led to a notable decrease in *CYP19A1* expression levels in these cells, in comparison to the siNC transfection (****p < 0.0001). The proliferation of HGC27 and AGS cells after disrupted *CYP19A1* expression was evaluated using the MTT assay. It was found that knocking down the expression of *CYP19A1* significantly inhibited the proliferative ability of HGC27 and AGS cells (Fig. 7F,G). In order to further verify the regulatory roles of *CYP19A1* in ER stress, we further detected the expression level of ER stress-related markers, including *ATF4*, *DDIT3* and *XBP1*, and found that the silence of *CYP19A1* expression also significantly decreased the expression of *ATF4*, *DDIT3* and *XBP1* (Fig. 7H,I). The results indicated that *CYP19A1* not only served as an ERSRG but also might regulate the ER stress-related pathways in GC cells.

## 4 Discussion

In the previous studies, ER stress has been classified as a major form of procedural cell death when excessive ER stress and sustained autophagy occur [33]. However, the malignant cells exploit the ER stress reaction pathway as an adapted survival mechanism to resist programmed cell death [33,34]. The specific underlying mechanisms of ERSRGs in STAD remain unclear. Furthermore, several drugs that can regulate ER stress-related pathways to promote cancer cell death have also been reported [35,36]. In addition to the previously mentioned drugs, there may be a more critical function of ERSRGs in the pathogenesis and progression of STAD. Consequently, in this study, the relationships between ERSRGs and STAD prognosis were thoroughly examined. The nomogram model of prognostic risk scores from the ERSRGs led to the establishment of novel prognostic biomarkers and curative therapeutic targets in STAD.

In this study, we first investigated the roles of ERSRGs in STAD using bioinformatics methodologies. Based on the differentiated expressed ERSRGs, a signature of STAD prognostic risk score was developed, which contained five prognostically relevant ERSRGs: *AKR1B1*, *SERPINE1*, *ADCYAP1*, *MATN3*, and *CYP19A1*. This signature model could serve as a valuable tool for STAD prognosis prediction and risk assessment. Besides, compared to models 1 and 2 from related studies, our model showed no significant difference in the AUC under the ROC curve for predicting the 1-year prognosis of patients with GC. However, the AUC for the 3-year and 5-year prognostic predictions was significantly better than that of models 1 and 2 [29,30,37]. In addition, we also constructed a nomogram to predict 1-, 3-, and 5-year survival in patients with STAD, enhancing its clinical applicability. The evaluation of the nomogram’s predictive ability through ROC curves also revealed that the ERSRGs prognostic risk score outperformed other clinical pathological indicators.

Meanwhile, studies have shown that the tumor immune microenvironment serves a crucial pivotal role in the onset and progression of solid cancers, including STAD, revealing a range of immune characteristics dependent on specific etiologies [38–40]. Evaluation of immune cells infiltration using the TCGA-STAD and GSE84437 databases demonstrated notable differences in T-cell subsets, such as CD8^+^ T cells, Tregs, Th1, macrophages, and DCs, between the high-risk and low-risk groups. This suggests that patients in the high-risk group may exhibit distinct immune suppression or activation states, potentially affecting their response to immunotherapy, such as immune checkpoint inhibitors (ICIs). Furthermore, differences in immune-related pathways between the high- and low-risk groups suggested that the high-risk group may exhibit stronger immune suppression signals, such as upregulated Tregs, which could lead to immune evasion and increased tumor resistance to ICIs immunotherapy. Additionally, immune checkpoint-related genes, including CD40, CD276, PD-L2, and TNFSF4, showed higher expression levels in high-risk patients, implying that they may be more suitable candidates for ICIs therapy. The classification of patients into high- and low-risk groups based on the model provides valuable predictive information for clinical applications. This stratification aids in personalized cancer immunotherapy, potentially improving treatment success rates [41–43].

scRNA-seq technology, a derivative of next-generation sequencing, has experienced rapid advancements in recent years [44,45]. Every tumor cell is characterized by unique somatic variations, transcriptional regulation, and epigenetic modifications. scRNA-seq is effectively utilized to analyze transcriptome heterogeneity and to identify rare cell types and states in various kinds of cancers. Beyond meticulously delineating the distinctions between malignant and non-malignant cells, scRNA-seq has the potential to identify specific cell subpopulations, biomarkers or tumor heterogeneity. This capability could significantly refine the existing classification of STAD, deliver precious insights into GC, and hopefully advance semiconductor biomedical research and clinical practice [46]. We obtained a comprehensive transcriptional profiling microarray from the GEO database, which allowed us to characterize 23,060 single cells from five surgically resected diffuse GCs obtained superficially and profusely, as well as normal cells matched on scRNA-seq. Among these selected five genes, in the univariate Cox regression analysis, the Hazard Ratio for *CYP19A1* is 2.107, much higher than *AKR1B1* (1.456), indicating that it has the greatest impact on prognosis. Secondly, although the coefficient value (coefgene) of *CYP19A1* (0.0692) in the model genes is lower than that of *SERPINE1* (0.071), ranking second, single-cell analysis revealed that *CYP19A1* is not expressed in normal gastric tissue. In contrast, its expression is elevated in deep GC tissues. However, *AKR1B1* and *SERPINE1* are expressed in both normal gastric tissue and GC tissues, which lack specificity. Therefore, we ultimately chose *CYP19A1* as the research target.

Previous studies have found that overexpression of *CYP19A1* contributed to the pathogenesis, progression, and distant organ metastasis of estrogen receptor-α-positive breast cancer [47,48]. Furthermore, Exemestane, letrozole, and anastrozole, as typical third-generation *CYP19A1* inhibitors, have been investigated in breast cancer treatment by targeting CYP19A1 [49–51]. Moreover, by targeting the inhibition of CYP19A1, Anastrozole also effectively reversed the chemoresistance of colorectal cancer cells [52,53]. In our study, single-cell analysis demonstrated that *CYP19A1* expression was undetectable in normal gastric tissue cells but was present in GC cells. Silencing the expression of *CYP19A1* markedly suppresses the proliferation of GC cells, which aligns with the findings reported by Zhou et al. [54,55]. Recent studies also demonstrated that exemestane, a novel chemotherapeutic agent for GC, inhibits GC cells’ proliferation by targeting CYP19A1. Meanwhile, the combination of exemestane and 5-fluorouracil (5-FU) offers a more effective therapeutic strategy for GC [56]. Besides, Hedyotis diffusa Willd ethanol extract, Ursolic acid (UA), exhibited significant cytotoxic activity against GC cells. Most importantly, molecular docking simulations using the 3D structure of CYP19A1 revealed an excellent fitting score for UA. UA could be used to treat GC by silencing the expression of CYP19A1. As a result, UA may serve as an effective CYP19A1 inhibitor [57]. All these findings indicated that targeting CYP9A1 may potentially yield significant clinical therapeutic benefits for patients with STAD.

Meanwhile, the RT-qPCR analysis revealed an upregulation of *CYP19A1* expression in GC cell lines. Furthermore, it was observed that the silence of *CYP19A1* expression in HGC27 and AGS STAD cell lines significantly hampered their proliferation ability and the expression of ER stress markers, including *ATF4*, *DDIT3* and *XBP1*. Besides, recent research has also shown that CYP19A1 can regulate ER stress to affect different cancer development. For example, by inducing ER stress in estrogen receptor-positive breast cancer cells, ionizing radiation downregulates CYP19A1 expression and reduces estradiol synthesis, which ultimately inhibits cell proliferation [58]. Furthermore, the *CYP19A1* gene encodes an enzyme that belongs to the cytochrome P450 superfamily [25]. This enzyme is responsible for aromatase expression and estrogen synthesis, regulated by hormones, cytokines, and immune modulators under both normal and pathophysiological conditions. Many studies have confirmed that estrogens could regulate ER stress through different mechanisms, a phenomenon that is widely observed in adipose tissue [28], brain tissue [26], and diseases related to breast tissue. More importantly, in breast cancer, estrogen secretion can cooperate with the UPR to cope with ER stress. Furthermore, this condition was later confirmed to be one of the mechanisms by which UPR promotes the development of breast cancer [27]. Overexpression of CYP19A1 contributed to the pathogenesis, progression, and distant organ metastasis of estrogen receptor-α-positive breast cancer [47,48]. Estrogen can also modulate protein folding and degradation pathways, and its action through estrogen receptors (ERα and ERβ) could also influence the expression of UPR-related genes [59,60]. Therefore, the silencing of CYP19A1 expression likely reduces estrogen production, which further affects the activation of the UPR and reduces the expression of ER stress-related markers such as ATF4, XBP1, and DDIT3. This point also indicated that CYP19A1 might play an important regulatory role in modulating the ER stress response and overall cellular homeostasis by affecting estrogen signaling in STAD cells.

However, despite rigorous analysis and extensive data validation, our study still with limitations. Firstly, this study is retrospective research and lacks prospective results. Additionally, a more comprehensive investigation at the protein level, along with *in vitro* and *in vivo* studies, is still warranted. At last, the precise molecular mechanisms of CYP19A1 action in STAD remain to be deeply elucidated.

## 5 Conclusion

Using bioinformatics, this study conducted a thorough assessment of ERSRGs in STAD and established a five-ERSRG risk score signature to assess the prognosis of patients with STAD. Furthermore, by integrating scRNA-seq data, the core gene *CYP19A1*, which was only expressed in tumor tissues and not expressed in normal tissues among these five selected genes, was identified. We further found that high expression of *CYP19A1* was related to a poor survival outcome for patients with STAD. Besides, compared to normal gastric epithelial cells, the expression of *CYP19A1* was significantly higher in STAD cell lines. Knockdown of the expression of *CYP19A1* significantly inhibited the cell proliferation ability and decreased the expression of ER stress-related genes, including *ATF4*, *DDIT3* and *XBP1*. In conclusion, our study developed a novel prognosis prediction signature and provided novel biomarkers and therapeutic targets for patients with STAD.

## Supplementary Materials





## Data Availability

The datasets generated and/or analyzed during the current study are available from the corresponding authors on reasonable request.

## Supplementary Materials

The supplementary material is available online at https://www.techscience.com/doi/10.32604/or.2025.062250/s1.

## Abbreviations

STAD: Stomach adenocarcinoma
ER: Endoplasmic reticulum
GC: Gastric cancer
UPR: Unfolded protein response
IRE1: Inositol-requiring enzyme 1
ATF6: Activating transcription factor 6
PERK: Protein kinase R-like ER kinase
ERSRGs: ER stress-related genes
CIP2A: Cancerous inhibitor of protein phosphatase 2A
XBP1: IRE1a-X-Box Binding Protein 1
PD1: Programmed death protein 1
CTLA4: Cytotoxic T lymphocyte-associated antigen 4
GRP78: Glucose-Regulated Protein 78
CHOP: Pro-apoptotic protein C/EBP homologous protein
CYP19A1: Cytochrome P450 Family 19 Subfamily A Member 1
scRNA-seq: single-cell RNA sequencing
siRNA: small interfering RNA
ATF4: Activating Transcription Factor 4
DDIT3: DNA Damage Inducible Transcript 3
TCGA: The Cancer Genome Atlas
DEGs: Differentially expressed genes
GEO: Gene Expression Omnibus
OS: Overall survival
LASSO: Least absolute shrinkage and selection operator
K-M: Kaplan-Meier
ROC: Receiver operating characteristic
FC: Fold change
GO: Gene Ontology
KEGG: Kyoto Encyclopedia of Genes and Genomes
ssGSEA: single-sample gene set enrichment analysis
MTT: 3-(4,5-dimethylthiazol-2-yl)-2,5-diphenyl tetrazolium bromide
RT-qPCR: Real-time quantitative polymerase chain reaction
ECM: Extracellular matrix
aDCs: activated Dendritic Cells
DCs: Dendritic Cells
iDCs: immature Dendritic Cells
Th1: Type 1 T helper
Th2: Type 2 T helper
TIL: Tumor-Infiltrating Lymphocytes
pDC: plasmacytoid dendritic cells
Tfh: T follicular helper cells
APC: Antigen-Presenting Cell
CCR: C Chemokine Receptor
HLA: Human leukocyte antigen
ICIs: Immune checkpoint inhibitors
t-SNE: t-Distributed Stochastic Neighbor Embedding
DMSO: Dimethyl sulfoxide
PCA: Principal component analysis
AKR1B1: Aldo-Keto Reductase Family 1 Member B1
SERPINE1: Serpin Family E Member 1
ADCYAP1: Adenylate Cyclase Activating Polypeptide 1
MATN3: Matrilin 3
AUC: Area Under the Curve
BP: Biological process
CC: Cellular component
MF: Molecular function
CMP: Common myeloid progenitor
BM: Bone Marrow
EIF2AK3: Eukaryotic Translation Initiation Factor 2 Alpha Kinase 3
HSPA5: Heat Shock Protein Family A (Hsp70) Member 5
ERN1: Endoplasmic Reticulum to Nucleus Signaling 1
5-FU: 5-fluorouracil
UA: Ursolic acid
