# Supplementary material for: CYP19A1 Silencing Inhibits Cell Proliferation and Endoplasmic Reticulum Stress in Stomach Adenocarcinoma
Source: Oncol Res. 2025 Sep 26;33(10):2937–60. doi: 10.32604/or.2025.062250 (PMC12493987; doi:10.32604/or.2025.062250)
Supplement: Supplementary file 1 [file OncolRes-33-62250-s001.docx]

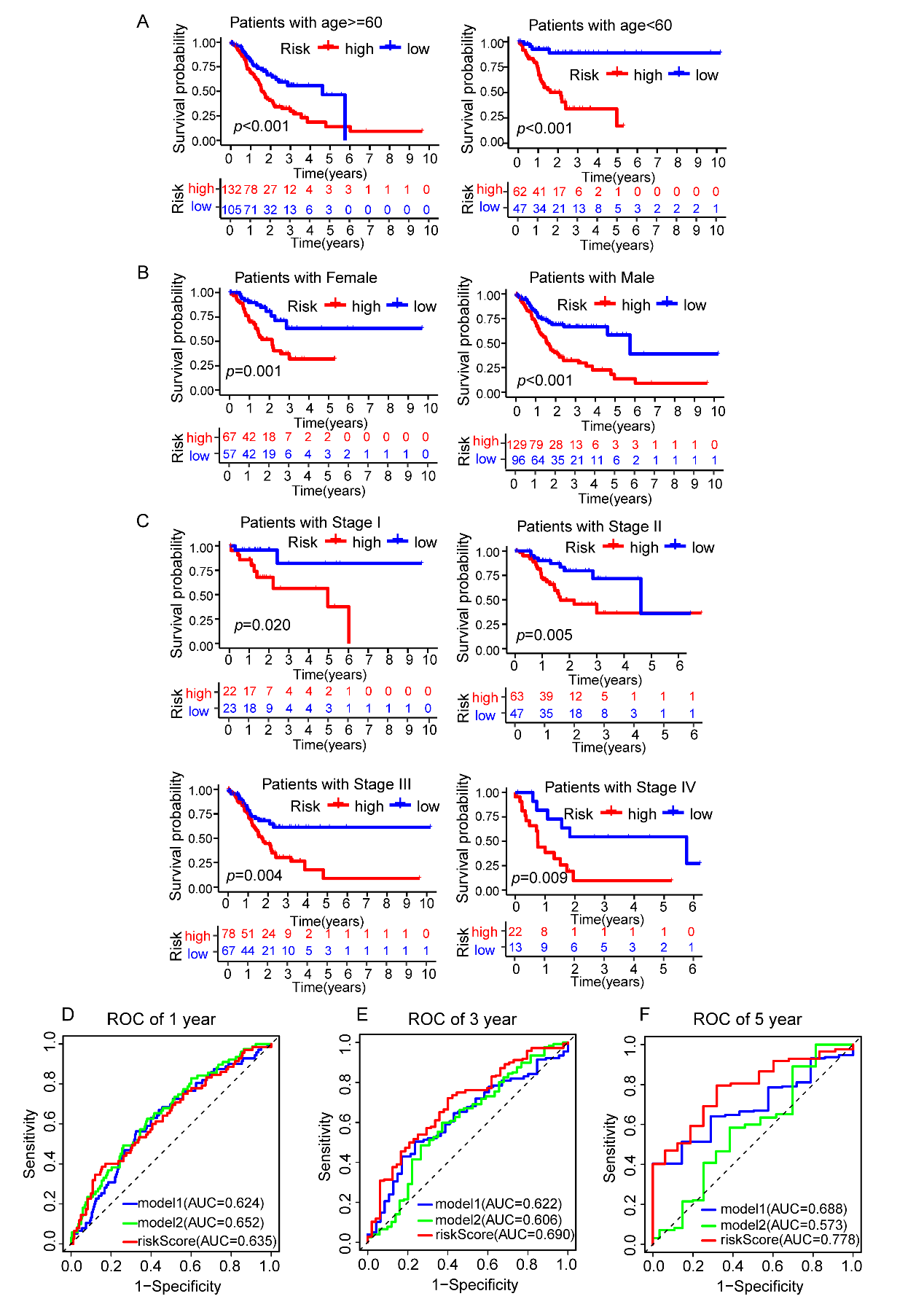


Figure S1. K-M survival curve among different age (A), sex (B) and stages (C) based on the TCGA-STAD data. Evaluation of the prognostic prediction capability of the ERSRGs risk score signature compared with model 1 and model 2 using the ROC curve based on the TCGA-STAD data in 1-year (D), 3-year (E), 5-year (F).


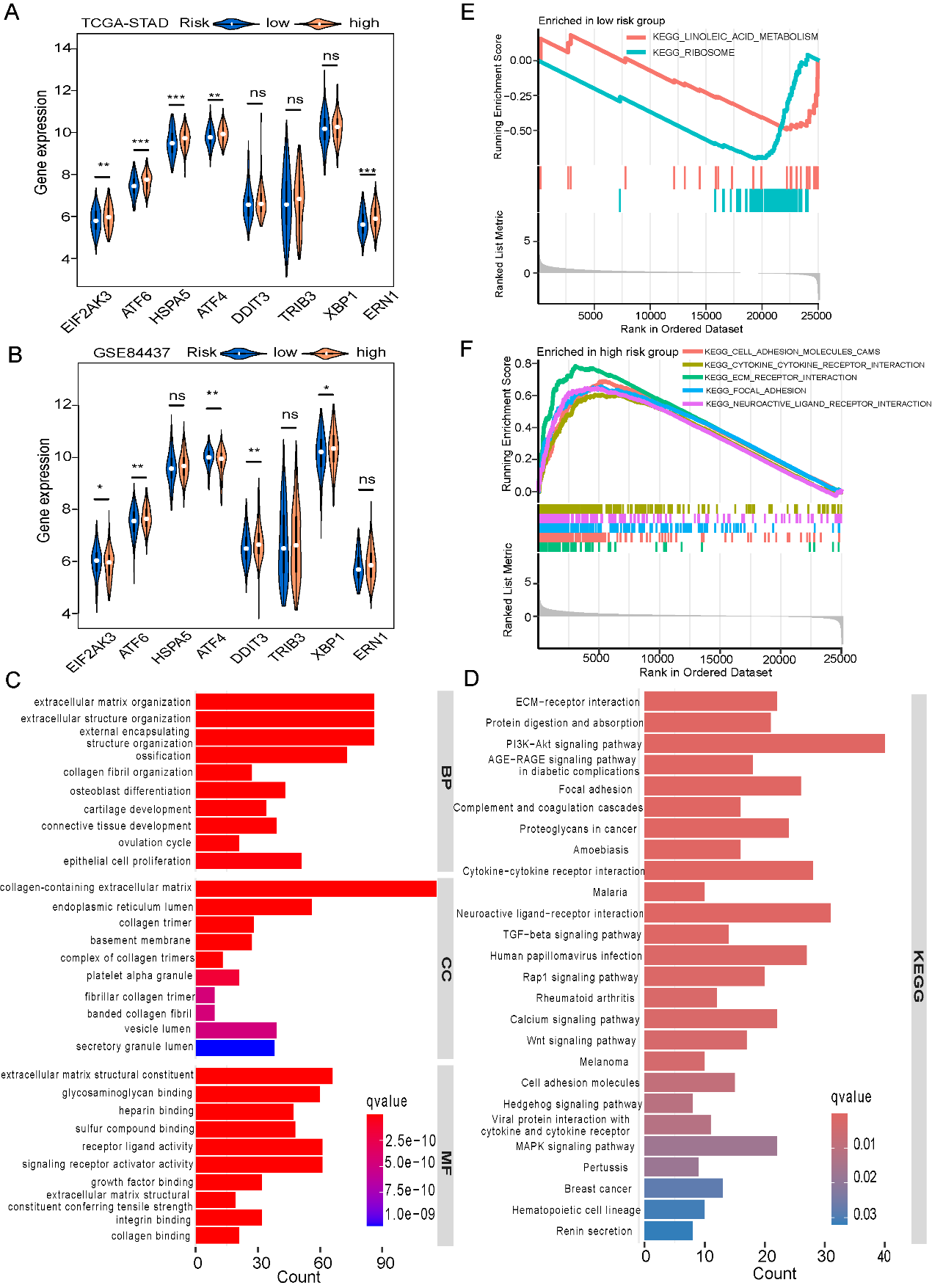


Figure S2. Function Enrichment Analysis Based on ERSRGs Risk Score Signature.

- 1. The expression level of the ER stress markers in the different risk groups based on the TCGA-STAD data;
  2. The expression level of the ER stress markers in the different risk groups based on the GSE84437 data;
  3. The GO enrichment analysis of the different risk groups based on the TCGA-STAD data;
  4. The KEGG pathway enrichment analysis of the different risk groups based on the TCGA-STAD data;
  5. The GSEA analysis in the low-risk group based on the TCGA-STAD data;
  6. The GSEA analysis in the high-risk group based on the TCGA-STAD data.

ns, no significance, * *p*<0.05, ** *p*<0.01, *** *p*<0.001


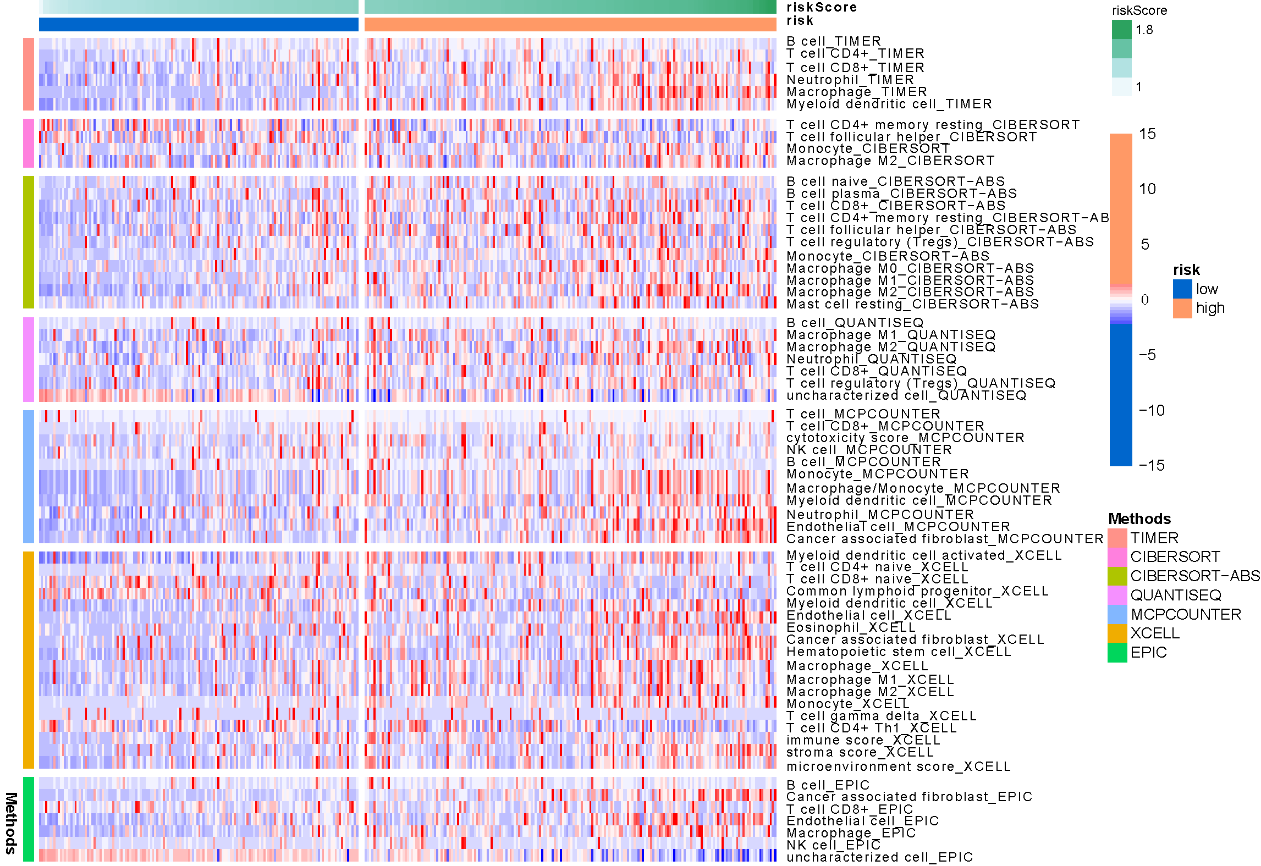


Fig S3. Heatmap of the difference in infiltrating immune cells between the high- and low-risk groups based on the TCGA-STAD data.


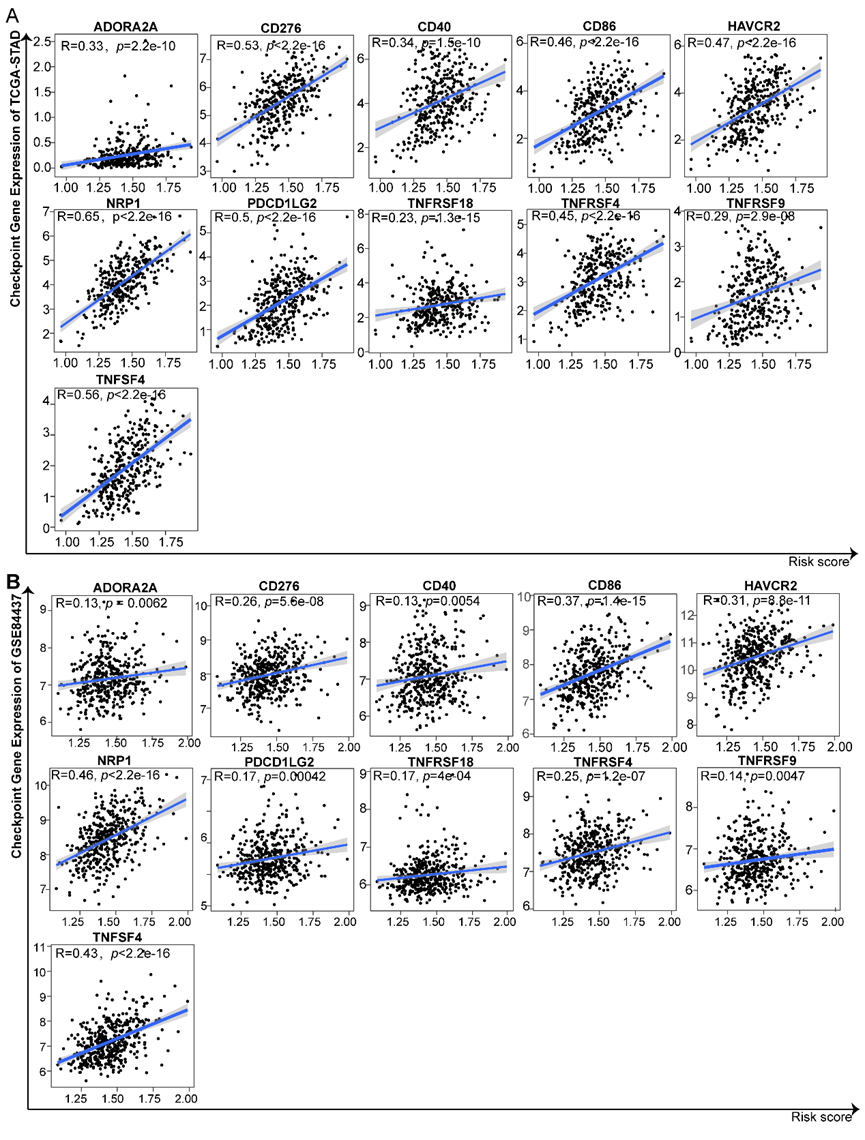


Figure S4. The expression of eleven immune checkpoint genes was positively correlated with the risk score in TCGA-STAD dataset (A) and GSE84437 dataset (B).


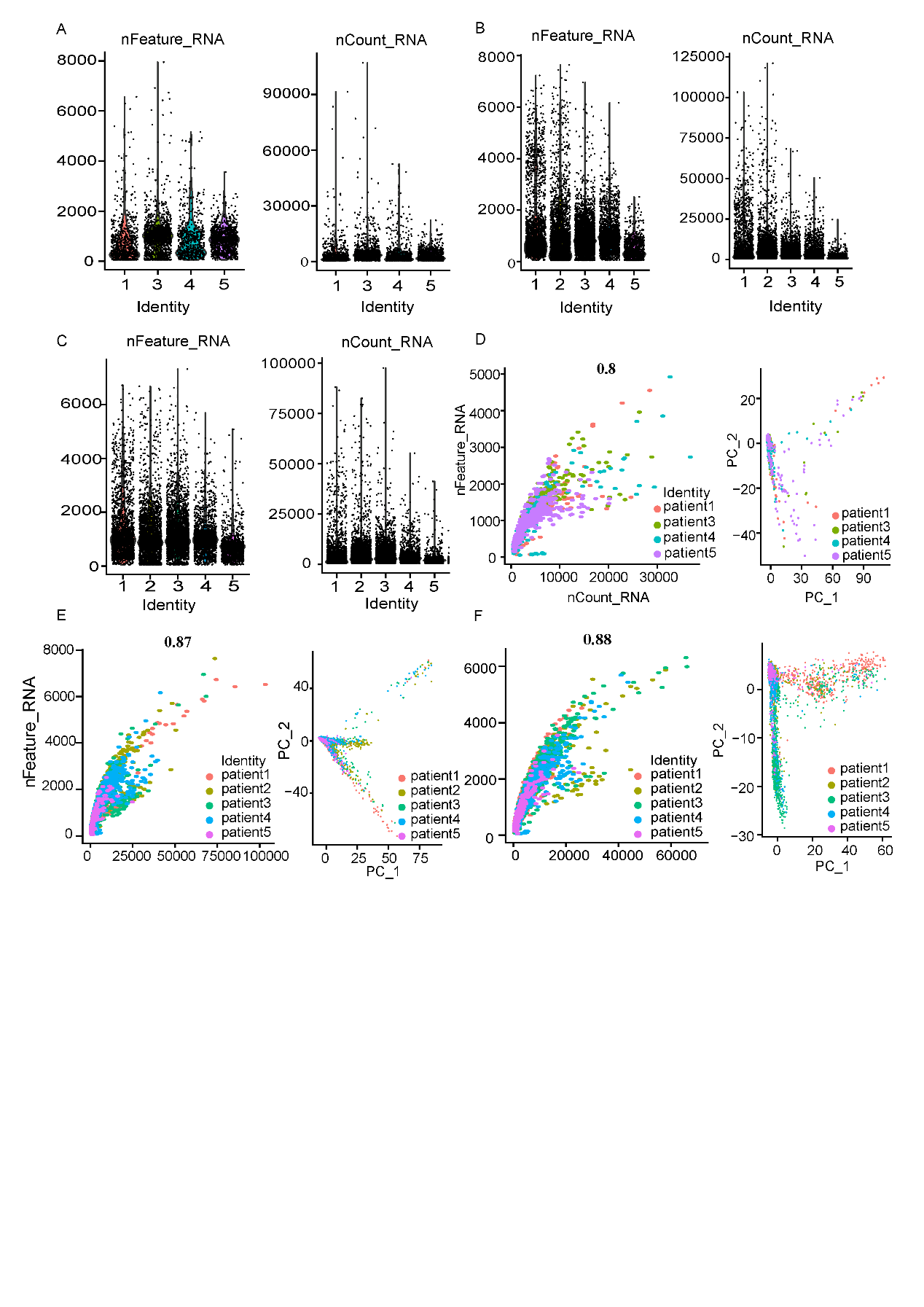
\

Figure S5. (A-C) Data filtering process of scRNA-seq analysis. (D-F) Analysis of sequencing depth and PCA.


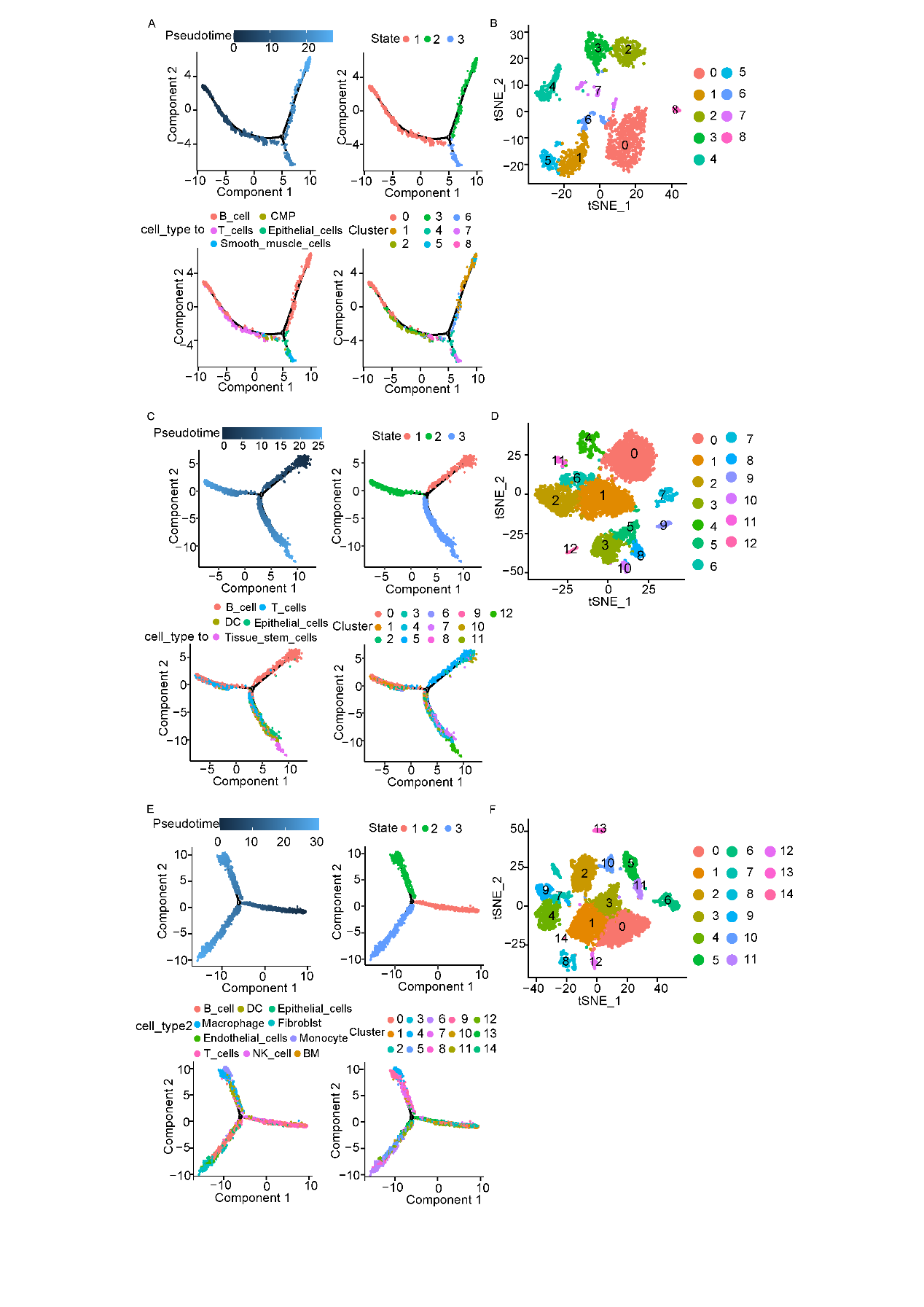


Figure S6. The results of cell trajectory analysis in normal gastric tissues, superficial layer and deep layer tissues of gastric cancer.

- 1. The cell trajectory analysis to trace the developmental pathways of normal gastric tissue.
  2. t-SNE clustering was utilized to categorize cells based on gene expression patterns in normal gastric tissues.
  3. The cell trajectory analysis to trace the developmental pathways of superficial layer tissues of GC.
  4. t-SNE clustering was utilized to categorize cells based on gene expression patterns in superficial layer tissues GC.
  5. The cell trajectory analysis to trace the developmental pathways of deep layer tissues of GC.
  6. t-SNE clustering was utilized to categorize cells based on gene expression patterns in deep layer tissues of GC.
